# Phytochemical Characterization and Anticancer Activity of *Clerodendrum chinense* Leaf Extract Against Breast and Cervical Cancer Cells

**DOI:** 10.3390/ijms26062729

**Published:** 2025-03-18

**Authors:** Chuda Chittasupho, Weerasak Samee, Supachoke Mangmool, Narainrit Karuna, Songyot Anuchapreeda, Siriporn Okonogi, Sirivan Athikomkulchai

**Affiliations:** 1 Department of Pharmaceutical Sciences, Faculty of Pharmacy, Chiang Mai University, Chiang Mai 50200, Thailand; chuda.c@cmu.ac.th (C.C.); okng2000@gmail.com (S.O.); 2 Center of Excellence in Pharmaceutical Nanotechnology, Faculty of Pharmacy, Chiang Mai University, Chiang Mai 50200, Thailand; songyot.anuch@cmu.ac.th; 3 Department of Pharmaceutical Chemistry, Faculty of Pharmacy, Srinakharinwirot University, Ongkharak, Nakhon Nayok 26120, Thailand; weerasak@g.swu.ac.th; 4 Department of Pharmaceutical Care, Faculty of Pharmacy, Chiang Mai University, Chiang Mai 50200, Thailand; supachoke.man@cmu.ac.th (S.M.); narainrit.k@cmu.ac.th (N.K.); 5 Department of Medical Technology, Faculty of Associated Medical Sciences, Chiang Mai University, Chiang Mai 50200, Thailand; 6 Department of Pharmacognosy, Faculty of Pharmacy, Srinakharinwirot University, Ongkharak, Nakhon Nayok 26120, Thailand

**Keywords:** anticancer, apoptosis, *Clerodendrum chinense*, HELA, MCF-7, ROS, verbascoside

## Abstract

Cancer remains a significant global health challenge, necessitating novel therapeutic interventions. *Clerodendrum chinense* leaf extract (CCL) has gained interest for its potential anticancer properties due to its bioactive composition. This study aims to evaluate the cytotoxic effects of CCL against MCF-7 breast cancer and HeLa cervical cancer cells and elucidate its mechanisms of action. High-performance liquid chromatography identified verbascoside, isoverbascoside, and hispidulin as the major bioactive compounds. CCL exhibited time- and dose-dependent cytotoxicity, with MCF-7 cells showing greater sensitivity (IC_50_ = 126.8 µg/mL, 72 h) than HeLa cells (216.1 µg/mL, 72 h). Flow cytometry confirmed apoptotic induction, with late apoptosis increasing at moderate concentrations (16.03–23.55%) and necrosis prevailing at higher doses (50.80–63.68%). Reactive oxygen species generation was significantly elevated in MCF-7 (70.2%) and HeLa (60.4%) cells at 250 µg/mL. CCL effectively suppressed colony formation and cell migration in a dose-dependent manner. Molecular docking studies demonstrated that apoptosis induction of CCL bioactive compounds may mediate through the pro-apoptotic BCL2 associated X, apoptosis regulator (BAX) regulator. These findings highlight the potential of CCL as a natural anticancer agent with multiple mechanisms, including reactive oxygen species (ROS)-induced apoptosis, BAX activation, and inhibition of proliferation and metastasis.

## 1. Introduction

Cancer remains a significant global health burden, with an estimated 20 million new cancer cases and almost 10.0 million cancer deaths, particularly female breast and cervical cancers [1]. This drives the important need for innovative therapeutic strategies that are effective, affordable, and safe to tackle cancer prevalence. Current cancer treatments, such as chemotherapy and targeted therapies, are often associated with severe side effects, drug resistance, and high costs, highlighting the importance of exploring alternative and complementary approaches [2]. Among these, natural products derived from medicinal plants have emerged as promising new anticancer candidates for cancer therapy, offering diverse bioactive compounds with potential therapeutic efficacy, fewer side effects, and cost-effective solutions [3]. Plants have historically been a major source of natural product drug discovery, particularly in cancer treatment. Available plant-derived agents such as vinblastine, vincristine, etoposide, paclitaxel, docetaxel, topotecan, and irinotecan have already been highlighted as effective chemotherapeutics [4]. In addition, plant extracts rich in flavonoids, terpenoids, and phenolic acids exhibit potent anticancer properties by inducing apoptosis, modulating oxidative stress, and inhibiting cancer cell proliferation and metastasis [5,6]. *Clerodendrum chinense* (*C. chinense*), a traditional medicinal plant, is widely used in various cultures and has gained attention for its therapeutic potential in cancer treatments [7,8]. Phytochemical studies have identified a range of bioactive compounds in *C. chinense*, including flavonoids, terpenoids, and phenolic acids, which are linked to its pharmacological activities [8]. *C. chinense* has exhibited significant insecticidal effects, particularly in relation to the control of schistosomiasis and trichomoniasis [9]. Evidence is mounting on using *C. chinense* as an anticancer treatment; this aligns with up to 60% of anticancer candidates in clinical use that exhibited significant efficacy are natural product derivatives [10]. Leaf extract from *C. chinense* demonstrated potent anticancer activity against A549 lung cancer cells via inducing apoptosis and necrosis, enhancing reactive oxygen species (ROS) production, suppressing colony formation, and inhibiting lung cancer cell migration [11]. Recently, studies of *C. chinense* extract from stems and flowers illustrated substantial anticancer effects against MCF-7 breast cancer cells and HeLa cervical cancer cells [7,12].

Despite these promising findings, the comprehensive anticancer potential of *C. chinense* leaf extract (CCL), particularly its effects on breast and cervical cancer cell lines, remains underexplored. To underline the importance of bioactive compounds in *C. chinense* extract from different parts, it has been reported that the components of bioactive compounds in *C. chinense* could have different amounts of phytochemical compositions depending on the plant part [7]. Previous studies on *C. chinense* have reported anticancer effects primarily attributed to flavonoids, terpenoids, and phenolic acids [8]. Notably, CCL and its bioactive compounds, including verbascoside, isoverbascoside, and hispidulin, have shown cytotoxicity against various cancer cell lines, suggesting their therapeutic relevance [7,11,12]. However, limited studies have examined their effects on breast and cervical cancer cells. Incidence of breast and cervical cancers are increasing at an alarming rate; this study, therefore, aimed to investigate the anticancer activity of CCL against MCF-7 (breast cancer) and HeLa (cervical cancer) cell lines, focusing on its cytotoxicity, ROS generation, inhibition of colony formation, and migration.

The hypothesis of this work is that CCL contains bioactive compounds capable of inducing cancer cell death through both apoptotic and necrotic pathways and disrupting processes critical for tumor progression. By addressing these mechanisms, this study aims to bridge the gap in understanding the therapeutic potential of *C. chinense*, highlighting its role as a natural anticancer agent with broad-spectrum activity.

## 2. Results

### 2.1. Quantification and HPLC Analysis of Bioactive Compounds in CCL

**Figure 1:**
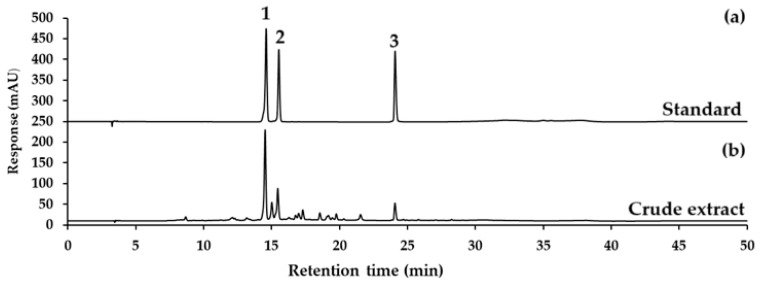
The HPLC chromatograms of (**a**) verbascoside (1), isoverbascoside (2), and hispidulin (3) standards; (**b**) CCL. All detected at a wavelength of 334 nm UV.

The quantification of the bioactive compounds in CCL was performed using HPLC-UV at a detection wavelength of 334 nm. The results are summarized in Table 1, showing verbascoside, isoverbascoside, and hispidulin contents for each sample, expressed as mean ± SD (µg/mL), along with the relative standard deviation (%RSD). The representative chromatograms for verbascoside, isoverbascoside, hispidulin standard, and CCL are displayed in Figure 1.

CCL showed the highest concentration of verbascoside at 900.57 ± 24.67 µg/mL, with a %RSD of 2.74%. Isoverbascoside was present at a lower concentration, with a mean of 259.22 ± 6.78 µg/mL and a %RSD of 2.61%. Hispidulin exhibited the lowest concentration among the three derivatives, with a mean of 64.62 ± 1.40 and a %RSD of 2.17 (Table 1). The relatively low %RSD values (<3%) across all compounds indicate good reproducibility of the analytical method.

The results indicate that verbascoside is the predominant compound in CCL, followed by isoverbascoside and hispidulin. This high content demonstrates the potential of the ethanolic extraction process for enriching verbascoside. The chromatographic separation was achieved with high resolution, as evident in the sharp and well-separated peaks observed in the chromatograms (Figure 1). The use of a C_18_-AR column and the optimized gradient elution conditions contributed to the efficient separation and detection of verbascoside at 334 nm. The %RSD values for all replicates across the samples were below 5%, indicating the method’s precision and reproducibility.

### 2.2. Cytotoxicity and Selectivity of CCL and Bioactive Compounds Against Cancer Cells

**Figure 2:**
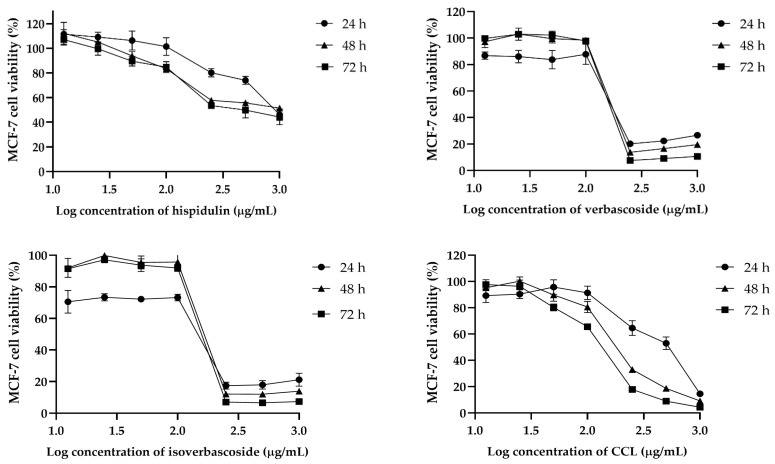
Effects of CCL and its bioactive compounds at various concentrations on the viability of MCF-7 cells. Data are expressed as Mean ± SD (*n* = 3).

**Figure 3:**
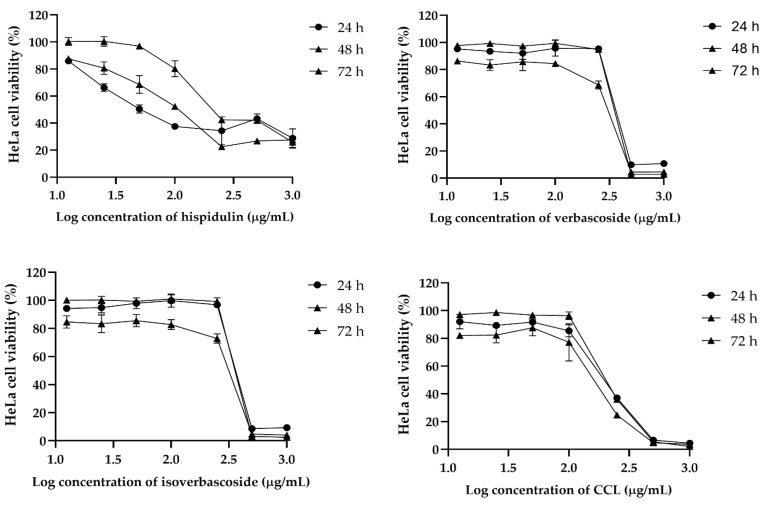
Effects of CCL and its bioactive compounds at various concentrations on the viability of HeLa cells. Data are expressed as Mean ± SD (*n* = 3).

**Figure 4:**
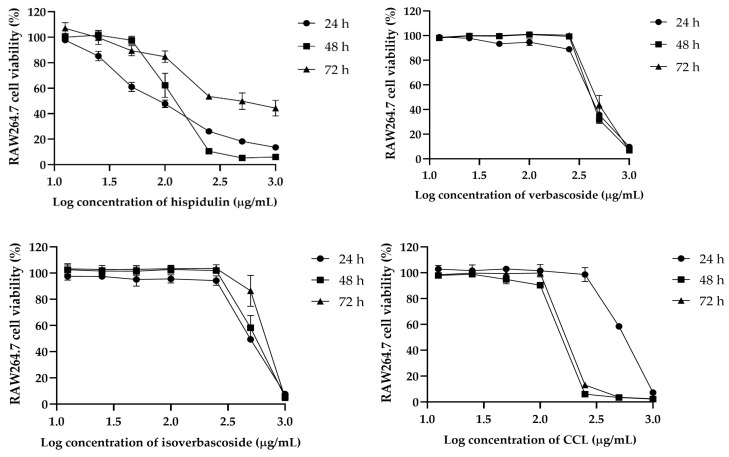
Effects of CCL and its bioactive compounds at various concentrations on the viability of RAW264.7 cells. Data are expressed as Mean ± SD (*n* = 3).

The cytotoxic effects of hispidulin, verbascoside, isoverbascoside, and CCL were assessed in MCF-7, HeLa, and RAW264.7 cells over 24, 48, and 72 h. In MCF-7 cells, isoverbascoside exhibited the highest cytotoxicity, with a sharp decline in viability at concentrations above 2.2 µg/mL, followed by verbascoside and CCL, while hispidulin showed a gradual and weaker effect (Figure 2). In HeLa cells, hispidulin showed dose-dependent cytotoxicity, whereas verbascoside and isoverbascoside also caused a substantial drop in viability at higher concentrations (Figure 3). In RAW264.7 cells, hispidulin induced a dose-dependent reduction in viability, while verbascoside, isoverbascoside, and CCL showed a sharp decline beyond a threshold concentration (Figure 4). These findings suggest that isoverbascoside, verbascoside, and CCL possess strong cytotoxic potential, particularly against breast cancer cells, while hispidulin and CCL had a relatively stronger cytotoxic effect against cervical cancer cells.

In MCF-7 cells, CCL exhibited a time-dependent decrease in IC_50_ values, from 425.6 µg/mL at 24 h to 126.8 µg/mL at 72 h, indicating enhanced cytotoxicity with prolonged exposure. Hispidulin showed the highest initial IC_50_ value (905.2 µg/mL at 24 h), which decreased significantly to 527.2 µg/mL at 72 h. Verbascoside and isoverbascoside exhibited relatively lower IC_50_ values, with verbascoside declining slightly from 185.6 to 173.3 µg/mL and isoverbascoside showing a minor fluctuation from 113.2 to 156.2 µg/mL over 72 h (Table 2).

For HeLa cells, a biphasic response was also observed in CCL that displayed an initial decline in IC_50_ from 196.7 µg/mL at 24 h to 152.7 µg/mL at 48 h, followed by an increase to 216.1 µg/mL at 72 h. Hispidulin exhibited an increasing IC_50_ value over time, from 98.18 µg/mL at 24 h to 311 µg/mL at 72 h. Similarly, verbascoside and isoverbascoside showed fluctuating IC_50_ values, with verbascoside increasing from 372.6 µg/mL at 24 h to 341.8 µg/mL at 72 h, and isoverbascoside showing a slight variation between 377 and 376.3 µg/mL over the same period (Table 2).

In RAW 264.7 macrophages, the IC_50_ values were higher compared to cancer cell lines. CCL showed an initial IC_50_ of 542.7 µg/mL at 24 h, decreasing to 151 µg/mL at 48 h and slightly increasing to 195.3 µg/mL at 72 h. Hispidulin exhibited a significant increase from 99.18 µg/mL to 180.2 µg/mL over 72 h, while verbascoside and isoverbascoside showed an increase in cytotoxicity but to a lower extent, indicating lower sensitivity of macrophages to these compounds (Table 2).

The IC_50_ values obtained for CCL and its bioactive compounds (hispidulin, verbascoside, and isoverbascoside) against MCF-7, HeLa, and RAW 264.7 cells revealed significant differences in cytotoxicity over time. The cytotoxicity of CCL may result from the combined effects of these three compounds, with isoverbascoside being the most potent in MCF-7 and hispidulin showing the highest activity in HeLa and RAW 264.7 cells.

The relative toxicity comparison assessed the cytotoxic selectivity of hispidulin, verbascoside, isoverbascoside, and the crude extract (CCL) by calculating the toxicity comparison ratios (IC_50_ of RAW 264.7/IC_50_ of MCF-7 and IC_50_ of RAW 264.7/IC_50_ of HeLa) at 24 h, 48 h, and 72 h post-treatment. The results revealed whether the compounds preferentially target cancer cells (MCF-7, HeLa) over RAW 264.7 macrophage-like cells (Table 3).

Isoverbascoside exhibited the highest tumor selectivity, with toxicity comparison ratios (IC_50_ of RAW 264.7/IC_50_ of MCF-7) of 4.41, 3.08, and 4.17, indicating that it was over four times more toxic to MCF-7 than to RAW 264.7 cells. Verbascoside also showed strong selectivity, with ratios of 2.3, 2.4, and 2.8, confirming its preferential toxicity toward MCF-7 cells but to a lesser extent than isoverbascoside. Hispidulin demonstrated the lowest selectivity, with ratios below 1.0, indicating that it was less toxic to MCF-7 than RAW 264.7 cells. CCL showed moderate selectivity, with fluctuating toxicity comparison ratios (1.3, 0.8, and 1.5), suggesting a moderate tumor-targeting effect.

For HeLa cells, isoverbascoside again exhibited the highest selectivity, with toxicity comparison ratios of 1.3, 1.8, and 1.7, confirming its preferential cytotoxicity toward HeLa but with lower selectivity than for MCF-7. Verbascoside showed moderate selectivity, with ratios of 1.2, 1.5, and 1.4, while hispidulin displayed minimal selectivity (1.0, 1.0, and 0.6), indicating similar cytotoxicity in RAW 264.7 and HeLa cells. CCL exhibited moderate selectivity, with ratios of 2.8, 1.0, and 0.9, showing higher selectivity at 24 h but a decline over time.

### 2.3. Annexin V-FITC/PI for Apoptosis Detection

**Figure 5:**
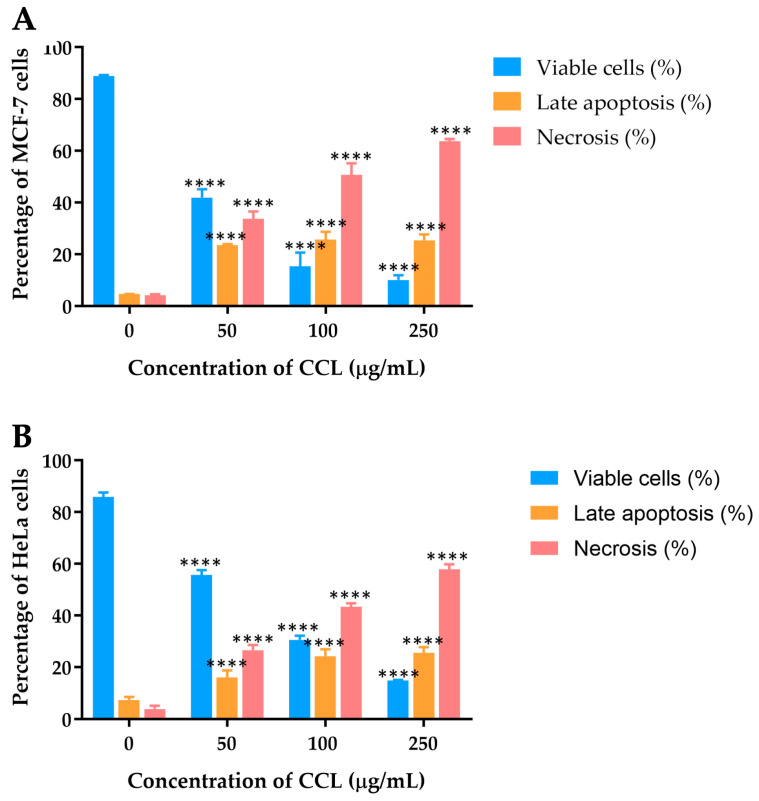
Apoptosis and necrosis induction by CCL in (**A**) MCF-7 and (**B**) HeLa cells at different concentrations. (n = 3) Data are expressed as Mean ± SD. All experiments were conducted in three independent replicates. **** *p* < 0.0001 indicates statistically significant differences compared with control (0 µg/mL).

The effects of CCL on MCF-7 and HeLa cell lines were further analyzed using Annexin V-FITC/PI staining, followed by flow cytometric analysis to differentiate viable, apoptotic, and necrotic cells. Figure 5 depicts the dose-dependent distribution of cell populations for CCL.

For MCF-7 cells treated with CCL, a marked reduction in viability was observed as the concentration increased (Figure 5A). At 50 µg/mL, the percentage of viable cells decreased to 41.95 ± 3.23%, with necrotic cells accounting for the majority of the population at higher concentrations. At 250 µg/mL, necrosis became the predominant mechanism of cell death, constituting over 63.68 ± 0.91% of the cell population. Late apoptosis was observed but remained secondary to necrosis across all concentrations.

In HeLa cells, CCL treatment also resulted in a dose-dependent decline in cell viability (Figure 5B). At 50 µg/mL, necrosis accounted for 26.63 ± 2.02% of the cell population, rising to over 57.95 ± 1.92% at 250 µg/mL. Late apoptosis was evident at all concentrations but remained less prominent than necrosis.

### 2.4. Molecular DOCKING of Verbascoside, Isoverbacisude, and Hispidulin with BAX, an Apoptosis Regulator

**Figure 6:**
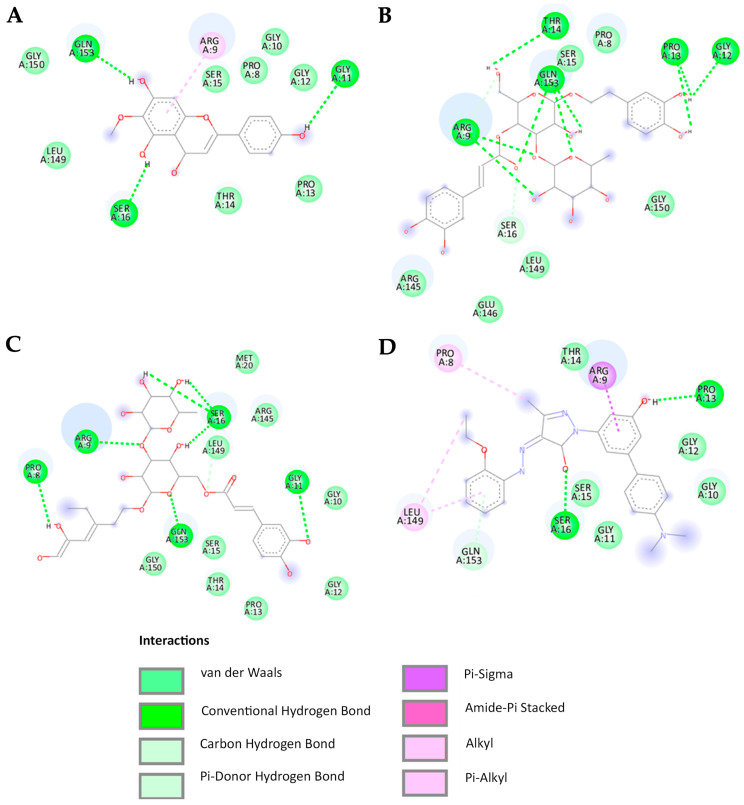
Molecular docking simulation results indicate that binding of (**A**) BAX activator (BTC-8), (**B**) verbascoside, (**C**) isoverbascoside, and (**D**) hispidulin to BAX protein.

**Figure 7:**
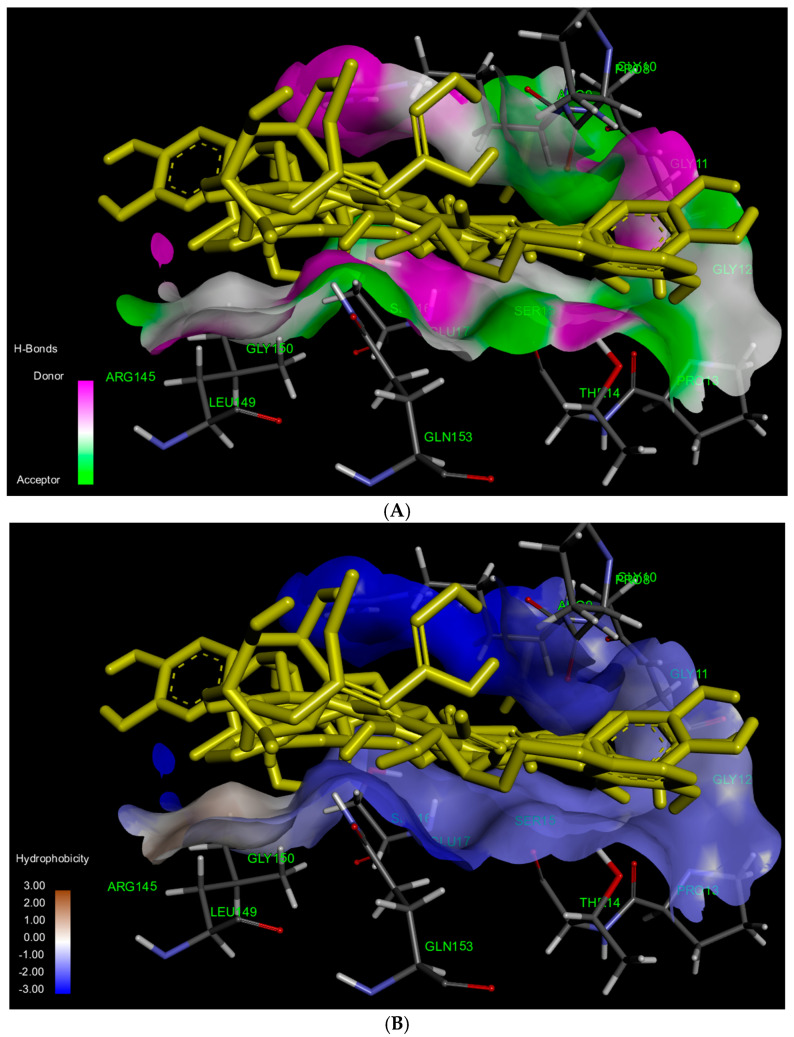
The results of the 3D molecular docking simulation highlighting (**A**) the hydrogen bond interactions and (**B**) the hydrophobic interactions observed in the superimposition of the BAX activator (BTC-8), verbascoside, isoverbascoside, and hispidulin with the BAX protein.

Regarding previous studies, bioactive compounds within CCL, particularly hispidulin, can up-regulate the ratio of BAX/Bcl-2 [13,14]. We, therefore, sought to study the interaction of bioactive compounds of CCL and BAX protein, supporting mechanistic insight. To explain cytotoxicity properties in our study, molecular docking was performed to assess the binding affinity of verbascoside, isoverbascoside, and hispidulin to the BAX protein (PDB: 1F16). BAX plays a crucial role in apoptosis by promoting mitochondrial outer membrane permeabilization (MOMP), leading to cytochrome c release and subsequent activation of caspases [15]. We assessed key docking parameters, including binding energy and inhibition constant (Ki), providing the strength and stability of these interactions. The docking was performed on the 1F16 crystal structure of BAX using a grid box size of 50 × 50 × 50, centered at X = 19.937, Y = 5.318, Z = 1.086 (Figure 6). The reference BAX activator exhibited a binding energy of -7.64 kcal/mol with a Ki of 2.53 µM, serving as a control for comparison. Among the tested compounds, hispidulin showed the highest binding affinity (−6.35 kcal/mol), followed by isoverbascoside (−4.94 kcal/mol) and verbascoside (−4.48 kcal/mol) (Table 4). Most compounds establish hydrogen bonds with the BAX protein at the amino acids Gly11, Pro13, Ser16, and Gly153 while engaging in Van der Waals interactions with the amino acids Gly10, Gly12, Thr14, Ser15, and Leu149 (Figure 7).

According to the molecular docking analysis, the BAX activator exhibited strong binding affinity with key amino acids, forming conventional hydrogen bonds with SER16 and PRO13, Van der Waals interactions with GLY10, GLY11, GLY12, THR14, and SER15, Pi-alkyl interactions with PRO8 and LEU149, Pi-donor hydrogen bonding with GLY153, and a Pi-sigma interaction with ARG9. Verbascoside interacted with ARG9, GLY12, PRO13, THR14, and GLN153 through conventional hydrogen bonds, while Van der Waals forces were detected with PRO8, SER15, ARG145, GLU146, LEU149, and GLY150. Additionally, a carbon/hydrogen bond was observed with SER16, indicating notable binding stability. Isoverbascoside formed conventional hydrogen bonds with PRO8, ARG9, GLY11, SER16, and GLN153, while Van der Waals interactions were observed with GLY10, GLY12, PRO13, THR14, SER15, MET20, and GLY150, suggesting a stable binding conformation. Hispidulin exhibited conventional hydrogen bonding with GLY11, SER16, and GLY153, along with Van der Waals interactions with PRO8, GLY10, GLY12, PRO13, THR14, SER15, LEU149, and GLY150 [16,17,18].

SER16, PRO13, and GLN153 play crucial roles as amino acids in the formation of conventional hydrogen bonds with ligands. The involvement of pi-alkyl or pi-sigma interactions with ARG9 is essential for promoting strong binding to the BAX protein, a relationship particularly exemplified by the high binding affinity observed with BTC-8 and hispidulin. In contrast, verbascoside and isoverbascoside exhibit lower binding affinities despite forming conventional hydrogen bonds with ARG9. This observation suggests that ligands characterized by a higher hydrophobic nature demonstrate enhanced affinity for BAX.

### 2.5. CCL Induces ROS Generation in MCF7 and HeLa Cells

The mechanism underlying the cell apoptosis induction of CCL was investigated. The ROS levels in MCF-7 and HeLa cells treated with and without CCL at different concentrations were measured using the DCFH-DA ROS detection assay kit. ROS formation was assessed in MCF-7 and HeLa cells treated with CCL at varying concentrations. A dose-dependent increase in ROS production was observed in both cell lines.

**Figure 8:**
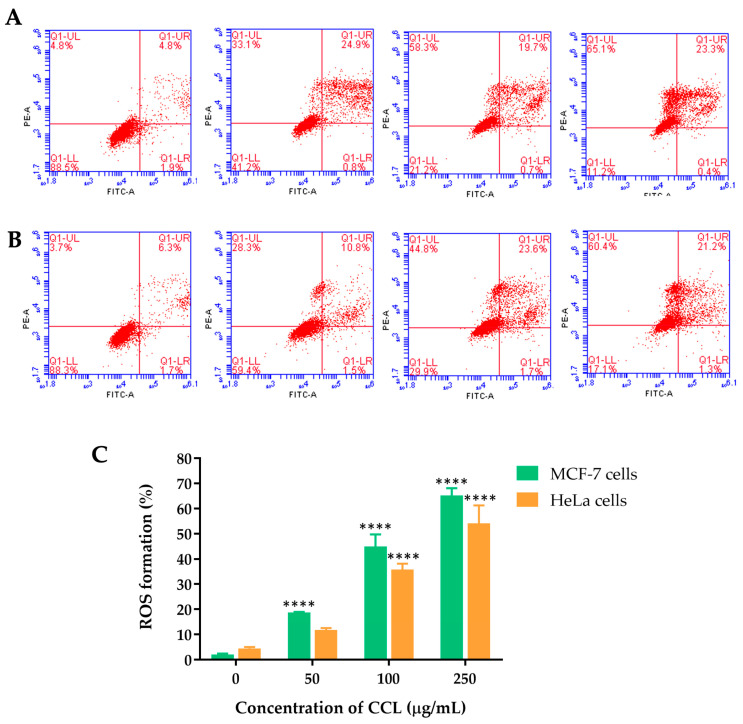
ROS formation induced by CCL in (**A**) MCF-7 and (**B**) HeLa cell lines, and (**C**) quantitative analysis of ROS formation. Data are expressed as Mean ±SD (n = 3). All experiments were conducted in three independent replicates. **** *p* < 0.0001 indicates statistically significant differences compared with control (0 µg/mL).

For CCL-treated cells (Figure 8), ROS formation in MCF-7 cells increased significantly, reaching approximately 65.23 ± 2.88% at 250 µg/mL. Similarly, ROS levels in HeLa cells rose to around 54.08 ± 7.15% at the same concentration. The data indicate that CCL induces oxidative stress more prominently in MCF-7 cells compared to HeLa cells.

### 2.6. CCL Decreases MCF-7 and HeLa Cells Colony Formation

**Figure 9:**
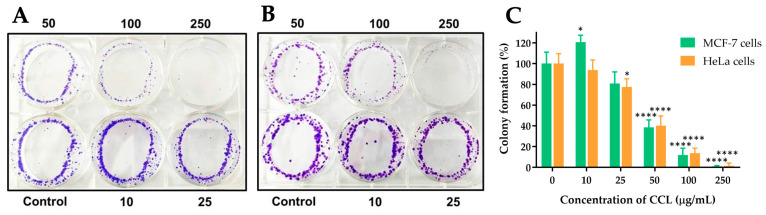
Effect of CCL on colony formation in (**A**) MCF-7 and (**B**) HeLa cells, and (**C**) quantitative analysis of colony formation. Data are expressed as Mean ± SD (n = 3). All experiments were conducted in three independent replicates. * *p* < 0.05 and **** *p* < 0.0001 indicate statistically significant differences compared with control (0 µg/mL).

The colony formation ability of MCF-7 and HeLa cells following treatment with CCL was evaluated and presented in Figure 6. CCL demonstrated a dose-dependent inhibition of colony formation in both cell lines (Figure 9A,B). At 10 µg/mL, colony formation was reduced by approximately 40% in MCF-7 cells and 30% in HeLa cells. Further reductions were observed at higher concentrations, with near-complete suppression at 250 µg/mL in both cell lines (Figure 9C).

### 2.7. Effect of CCL on the Migration of MCF-7 and HeLa Cells

The effects of CCL on the migration of MCF-7 and HeLa cells were evaluated using a scratch assay. The images and corresponding quantitative analysis demonstrate a concentration-dependent inhibition of cell migration in both cell lines after 48 h of treatment.

**Figure 10:**
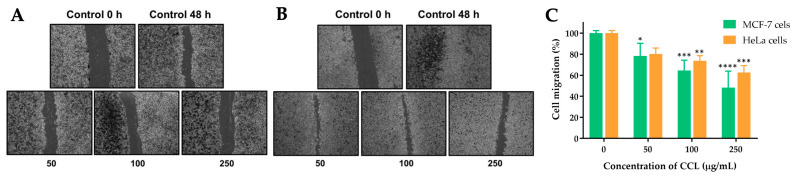
Effect of CCL on the migration of (**A**) MCF-7 and (**B**) HeLa cells, and (**C**) quantitative analysis of cell migration after 48 h. Data are expressed as Mean ± SD (n = 3). All experiments were conducted in three independent replicates. * *p* < 0.05, ** *p* < 0.01, *** *p* < 0.001, and **** *p* < 0.0001 indicate statistically significant differences compared with control (0 µg/mL).

For CCL-treated cells, MCF-7 cells showed a significant reduction in migration at concentrations of 50, 100, and 250 µg/mL. At the highest concentration (250 µg/mL), migration was reduced to approximately 48.35% of the untreated control (Figure 9). Similarly, in HeLa cells, migration was reduced to 62.73% at 250 µg/mL. Lower concentrations exhibited partial inhibition of migration, with 36% and 27% reduction observed in MCF-7 and HeLa cells, respectively, at 100 µg/mL (Figure 10).

## 3. Discussion

The benefits of *C. chinense* have been emerging for its anticancer activities based on different parts of *C. chinense* extract [7,11,12]. Identifying unique phytochemical compositions of *C. chinense* and their bioactivities against cancer cells are important processes in the context of developing anticancer treatments. In the present study, we found that verbascoside is the major bioactive constituent in the CCL, followed by isoverbascoside and hispidulin in a similar pattern to *C. chinense* flower extract [12]. However, only verbascoside and isoverbascoside are detected in *C. chinense* stem extract, while hispidulin is present in negligible amounts [7]. This underlines significant phytochemical composition differences and their bioactivities among *C. chinense* parts.

Our present study demonstrates that CCL exhibits potent anticancer activity against MCF-7 and HeLa cells through multiple mechanisms. These findings align with previous studies on *Clerodendrum* species, which have reported anticancer properties attributed to flavonoids, terpenoids, and phenolic acids [7,19]. IC_50_ values for CCL and its bioactive compounds—verbascoside, isoverbascoside, and hispidulin—varied across MCF-7, HeLa, and RAW 264.7 cell lines, indicating differential cytotoxicity over time. Isoverbascoside showed the highest potency in MCF-7 cells, while hispidulin exhibited the strongest activity in HeLa and RAW 264.7 cells. The sharp decline in IC_50_ values for MCF-7 over 72 h suggests higher sensitivity of breast cancer cells to CCL’s bioactive compounds. The lower IC_50_ values of hispidulin indicate its key role in CCL’s cytotoxic effects, particularly in MCF-7 cells. In contrast, HeLa cells displayed a biphasic response, with an initial drop in IC_50_ values at 48 h followed by an increase at 72 h, suggesting potential cellular adaptation or resistance mechanisms. These may involve altered drug metabolism, drug efflux pump overexpression, or apoptotic pathway dysregulation, enabling cancer cells to evade cell death [20]. While RAW 264.7 macrophages exhibited lower cytotoxicity than cancer cells, fluctuating IC_50_ values suggest prolonged exposure may induce cytotoxic effects.

In MCF-7 breast cancer cells, the percentage of viable cells significantly decreased with increasing concentrations of CCL, suggesting a strong cytotoxic effect of CCL on breast cancer cells. The percentage of cells undergoing late apoptosis increased steadily with concentration, indicating that at moderate doses, CCL activates apoptotic pathways. However, at higher concentrations (250 µg/mL), necrosis became the predominant form of cell death, suggesting that prolonged or high-dose exposure to CCL overwhelms cellular repair mechanisms and leads to uncontrolled cell death. A similar trend was observed in HeLa cervical cancer cells, where cell viability showed a dose-dependent reduction. Late apoptosis increased moderately at lower concentrations, but unlike MCF-7 cells, HeLa cells exhibited a more pronounced apoptotic response at lower doses and were more prone to necrosis in response to CCL treatment. The distinct responses between the two cell lines suggest differences in their cellular mechanisms related to apoptosis and necrosis processes [21,22]. It has been reported that cancer cell lines exhibit significant genetic heterogeneity due to clonal dynamics and continuous genomic instability. This variation influences gene expression patterns and drug sensitivity and response [23]. Different bioactive compounds may have varying effects on apoptotic cell death and necrosis in cancer cell lines, such as Hela and MCF-7 cells [22,24]. Notably, further studies are required to investigate the distinct apoptotic and necrotic responses in various cancer cell lines treated with CCL. Flow cytometric analysis allowed precise differentiation between viable, early apoptotic, late apoptotic, and necrotic cells, offering mechanistic insights into the cytotoxic effects of CCL. Apoptotic cell death is widely recognized as a beneficial process for both cancer prevention and treatment. Generally, apoptosis is triggered through one of two pathways: the extrinsic pathway, also known as the death receptor pathway, or the intrinsic pathway, also referred to as the mitochondrial pathway [25,26]. The apoptosis data correlate well with the MTT assay results, confirming that the observed reduction in cell viability is primarily due to apoptotic induction rather than nonspecific cytotoxicity. Furthermore, the apoptotic pathway activation was supported by molecular docking studies, which revealed strong interactions between CCL bioactive compounds and the BAX protein, a key regulator of mitochondrial apoptosis. These findings reinforce the conclusion that CCL promotes programmed cell death rather than simple cell lysis, highlighting its therapeutic potential. BAX is a pro-apoptotic member of the Bcl-2 family that regulates mitochondrial outer membrane permeabilization (MOMP), a key event in intrinsic apoptosis [27,28]. In contrast, necrosis is an uncontrolled form of cell death, often triggered by external factors, leading to cell swelling and rupture, releasing intracellular components into the surrounding environment, which can cause inflammation and potential damage to nearby tissues [29].

To investigate bioactive compounds of CCL-related apoptosis mechanism using molecular docking, we demonstrated that hispidulin may contribute to BAX-mediated apoptosis showing the lowest binding energy, suggesting a relatively strong interaction with the BAX protein, which could explain its broad cytotoxic effects and low SI in MCF-7 and HeLa cells. Conversely, isoverbascoside and verbascoside showed weaker BAX binding but exhibited a high SI in these cancer cell lines, indicating their potential as selective candidates for breast and cervical cancer treatment. Importantly, all three bioactive compounds from CCL interacted with key amino acids essential for BAX activation, including Gly11, Pro13, Ser16, and Gly153, suggesting their potential to promote BAX-mediated apoptosis [28]. However, differences in binding strength and the specific non-covalent interactions may influence the extent of BAX activation and apoptotic response. Hispidulin formed additional interactions with Leu149 and Gly150, which are critical for stabilizing the BAX active conformation. This could enhance its pro-apoptotic effect, leading to more pronounced cytotoxicity. In contrast, verbascoside and isoverbascoside primarily relied on hydrogen bonding and Van der Waals interactions, which may be less effective in promoting BAX activation. Additionally, based on their chemical structures, hispidulin is more hydrophobic (log P = 1.3), whereas verbascoside and isoverbascoside are more hydrophilic (log P = −0.5). Consequently, hispidulin exhibits a higher affinity for the hydrophobic binding site of BAX. Previous studies have shown that hispidulin induces apoptosis through BAX activation, leading to mitochondrial membrane potential (MMP) loss and cytochrome c release, as well as an increased BAX/Bcl-2 ratio in multiple cancer cell lines, including CNE-2Z, SMMC7721, PANC-1, and HT29 cells [14]. In addition, it has been shown that hispidulin triggers MMP loss, leading to cytochrome c release, a hallmark of BAX-mediated apoptosis. In another study, BAX activation via oligomerization has been detected in hispidulin-treated cells, supporting its role in apoptotic signaling [30]. Verbascoside promotes apoptosis by activating the STAT3/Wnt/-catenin pathway and modulating BAX, Bcl-2/p53 [31]. Additionally, isolated verbascoside from *Phlomis nissolii* demonstrated apoptotic induction in the breast cancer cell lines [32]. However, functional assays (e.g., cytochrome c release, caspase activation, mitochondrial depolarization) are required to assess specific roles in apoptosis processes. To support the benefits of BAX activator, BAX agonists SMBA1–SMBA3 bind to BAX, inhibit Ser184 phosphorylation, and promote oligomerization, leading to cytochrome c release and apoptosis. SMBA1 showed efficacy in mouse lung cancer xenografts, driving the development of optimized compounds like CYD-4-61 and GL0385, benefiting breast cancer cell treatment [33]. Other agonists, BAM-7 and BTSA1, demonstrated preclinical potential in glioblastoma and acute myeloid leukemia (AML) cell lines [27,34,35]. However, we acknowledge certain limitations in the present study. Experimental validation studies, such as direct protein measuring and a series of in vitro studies, are required to confirm the observation of bioactive compounds of CCL interacting with BAX protein.

ROS formation increased progressively with higher CCL concentrations in both cancer cells. MCF-7 cells exhibited higher ROS production compared to HeLa cells at all tested concentrations, with a particularly pronounced difference at 100 and 250 µg/mL. At 250 µg/mL, ROS formation reached approximately 65% in MCF-7 cells, while in HeLa cells, it was around 55%. These findings suggest that MCF-7 cells are more susceptible to oxidative stress induced by CCL compared to HeLa cells. The observed increase in ROS production aligns with the hypothesis that oxidative stress is a key mechanism underlying CCL-induced cytotoxicity. Elevated ROS levels can lead to cellular damage. This oxidative stress disrupts cellular homeostasis, shifting the balance toward apoptotic cell death [36]. The higher susceptibility of MCF-7 cells to ROS generation may be due to differences in their antioxidant defense mechanisms or mitochondrial function compared to HeLa cells. Moreover, other studies showed that isoverbascoside contains anti-inflammatory effects by blocking toll-like receptor 4 dimerization and antioxidant activity [37,38]. Colony formation of MCF-7 and HeLa cells declines in a concentration-dependent manner. MCF-7 cells initially exhibit a slightly higher colony-forming capacity compared to HeLa cells at lower concentrations (10 and 25 µg/mL). However, at concentrations of 50 µg/mL and above, both cell lines show substantial reductions, with near-complete inhibition at 250 µg/mL. This indicates that CCL effectively inhibits the clonogenic potential of both cancer cell lines. These findings suggest that CCL exerts significant cytotoxic effects, impairing the ability of cancer cells to proliferate and form colonies. The observed inhibition may be attributed to the induction of oxidative stress, apoptosis, and necrosis, as previously demonstrated in cell viability and ROS assays. The slightly higher sensitivity of HeLa cells at lower concentrations could be due to differences in their metabolic activity or cell cycle regulation compared to MCF-7 cells.

Cell migration is a fundamental process in cancer progression, particularly in tumor invasion and metastasis [39,40]. In the control group, a substantial reduction in the gap between cells is observed after 48 h, indicating a high intrinsic migratory ability of both cell lines. As the concentration of CCL increases, cell migration is progressively inhibited in a dose-dependent manner. At 50 and 100 µg/mL, partial inhibition of closure is evident, while at 250 µg/mL, the migration of both MCF-7 and HeLa cells is significantly reduced, with minimal gap closure observed. The results show a decline in cell migration with increasing CCL concentrations. MCF-7 and HeLa cells exhibit similar responses at lower concentrations, with migration rates around 80% at 50 µg/mL. However, at 100 and 250 µg/mL, migration inhibition is more pronounced, with migration rates dropping to approximately 60% and 40%, respectively. These results suggest that CCL effectively impairs cancer cell motility, which is a crucial benefit for preventing metastatic progression. In line with this, Bo Hei et al. reported that verbascoside can suppress the migration and invasion of human glioblastoma cells [41].

Research on *C. chinense* has demonstrated that its leaf, flower, and stem extracts exhibit differential cytotoxic effects, primarily due to variations in their bioactive compound composition. Our study aligns with previous findings that identify verbascoside, isoverbascoside, and hispidulin as key anticancer compounds in *C. chinense*. Research on comparable plant extracts, such as *Clerodendrum infortunatum* and *Clerodendrum viscosum*, has also reported strong antioxidant and prooxidant activities, reinforcing the role of polyphenolic compounds in mediating oxidative stress-induced cell death [42,43].

Taken together, these results show that verbascoside and its isomer, isoverbascoside, and hispidulin demonstrate significant cytotoxicity against breast adenocarcinoma (MCF-7) and cervical cancer (HeLa) cells. The present study supports our previous studies that provide the anticancer activities of *C. chinense,* and their phytochemical compositions have been demonstrated through its flower, stem, and leaf extracts [7,11,12]. Moreover, studies from others showed that verbascoside, isoverbascoside, and hispidulin demonstrated anticancer activities in various cancer types, highlighting avenues for developing cancer treatment from *C. chinense* extract [44,45,46,47,48]. While our findings demonstrate the anticancer potential of *C. chinense*, future studies should explore its combinatorial use with standard therapies, assess its efficacy in in vivo models, and investigate advanced drug delivery strategies. These research directions will provide a more comprehensive understanding of its therapeutic applicability and pave the way for potential clinical development.

## 4. Materials and Methods

Acetonitrile gradient grade for liquid chromatography was purchased from Merck (Darmstadt, Germany). Formic acid was purchased from Sigma-Aldrich (Burlington, Massachusetts, USA). Verbascoside, isoverbascoside and hispidulin were purchased from ChemFaces (Wuhan, China). MCF-1 (HTB-22™), HeLa cells (CCL-2™), and RAW264.7 cells (TIB-71^TM^) were obtained from the American Type Culture Collection (ATCC; Manassas, VA, USA). Dulbecco’s Modified Eagle Medium (DMEM), fetal bovine serum (FBS), 100 U/mL penicillin, 100 µg/mL streptomycin, trypsin-EDTA, and 3-(4,5-Dimethylthiazol-2-yl)-2,5-Diphenyltetrazolium Bromide (MTT) were sourced from Invitrogen (Carlsbad, CA, USA). Annexin V-FITC and propidium iodide (PI) solution (Cat No. 558547) were obtained from BD Biosciences (San Jose, CA, USA). 2′,7′-Dichlorodihydrofluorescein diacetate (DCFH-DA) (Cat No. D6883) and crystal violet powder (Cat No. C0775) were purchased from Sigma Merck KGaA (Darmstadt, Germany).

### 4.1. Plant Collection, Identification and Extraction

*C. chinense* leaves were sourced from Chiang Mai, Thailand, at the GPS coordinates 18°45′48.5″ N, 98°59′43.0″ E. Assistant Professor Sirivan Athikomkulchai verified the plant’s identity and a voucher specimen (SIRA003) was archived at the Faculty of Pharmacy, Srinakharinwirot University, Nakhon Nayok, Thailand. The leaves were thoroughly washed and then dried in a hot air oven at 60 °C for 24 h. The dried material was finely powdered using an electric grinder. A five-gram portion of the powdered sample was macerated in 50 mL of 95% ethanol for 72 h. The extraction process included filtration and was repeated twice more to maximize yield. The pooled extracts were separated from the solid residue via filtration, and ethanol was removed using a Buchi Rotavapor-R300 rotary evaporator (Flawil, Switzerland).

### 4.2. Determination of Verbascoside, Isoverbascoside, and Hispidulin in CCL Using HPLC-UV

To determine verbascoside, isoverbascoside, and hispidulin in CCL, we utilized High-Performance Liquid Chromatography-Ultraviolet (HPLC-UV) for phytochemical profiling, quality control, and correlation with biological activity. HPLC-UV provided a reliable and validated method for accurate quantification.

#### 4.2.1. Chromatographic Conditions

HPLC analysis was performed using an Agilent 1260 Infinity II system (Santa Clara, CA, USA), which included a quaternary pump, an autosampler, a multi-column thermostat, and a photodiode array detector. Chromatographic separation was conducted on an ACE 5 C18-AR column (4.6 × 250 mm, 5 µm) from Aberdeen, Scotland, with a Phenomenex C18 guard column (4 mm × 3 mm, 5 µm) from Torrance, CA, USA. The mobile phase consisted of 0.1% formic acid in ultrapure water (phase A) and 0.1% formic acid in acetonitrile (phase B). The gradient elution started at 5% phase B, gradually increasing to 40% over 20 min, followed by an increase to 80% within the next 10 min. The system was held at 80% phase B for 5 min before being decreased back to 5% over 5 min, with an additional 10-min equilibration at 5% phase B. The flow rate was maintained at 1 mL/min, with detection set at 334 nm. The column temperature was controlled at 25 °C, and the injection volume was 10 µL.

#### 4.2.2. Standard Solution Preparation

A stock solution of each standard was prepared at a concentration of 500 µg/mL in ethanol. Serial dilutions were then carried out to obtain standard solutions within the following concentration ranges: 5–50 µg/mL for verbascoside, 5–25 µg/mL for isoverbascoside, and 1–5 µg/mL for hispidulin.

#### 4.2.3. Sample Solution Preparation

A crude extract (75 mg) was dissolved in 1 mL of ethanol, achieving a concentration of 75 mg/mL. This solution was subsequently diluted 20-fold with 50% acetonitrile in 0.1% formic acid, resulting in a final concentration of 3.75 mg/mL. Further, a 10-fold dilution was performed using the same solvent system. Before injection, all prepared solutions were filtered through a 0.45 µm nylon membrane filter.

### 4.3. Cell Culture

MCF-7, HeLa, and RAW264.7 cells were cultured in DMEM supplemented with 10% FBS and 1% penicillin/streptomycin. Cell culture was maintained at 37 °C in a 5% CO_2_ incubator.

### 4.4. Cell Viability Assay

The assessment of cell viability was performed using an MTT assay to assess the cytotoxic effects of CCL on MCF-7, Hela, and RAW264.7 cells. Initially, MCF-7, HeLa, and RAW264.7 cells were seeded onto 96-well plates at a density of 2 × 10^4^ cells/mL. Following a 24-h incubation period, the culture medium was replaced with a serum-free medium containing specified concentrations of CCL, verbascoside, isoverbascoside, and hispidulin ranging from 12.5 to 1000 µg/mL. Following 24, 48, and 72 h of treatment, an MTT solution (0.5 mg/mL in culture medium) was added to each well and incubated at 37 °C for 2 h. The metabolically active cells converted MTT into formazan crystals, which were then dissolved in 100 µL of DMSO per well. Absorbance was recorded at 550 nm using a SpectraMax 3 microplate reader (Molecular Devices, San Jose, CA, USA). The IC_50_ values were determined using GraphPad Prism v.7.0 (La Jolla, CA, USA). Cell viability was assessed in comparison to untreated control cells using the following equation:

$$\mathrm{Cell}\;\mathrm{viability}\;\left(\%\right)=\frac{A550\mathrm{sample}}{A550\mathrm{control}}\times 100\%$$

### 4.5. Apoptosis Analysis

Apoptosis analysis was performed to evaluate the effects of CCL in inducing programmed cell death in cancer cells through apoptotic mechanisms. Briefly, flow cytometry was used to quantitatively differentiate between live, early apoptotic, late apoptotic, and necrotic cells, providing a mechanistic understanding of cell death beyond simple viability assays. MCF-7 and HeLa cells (2.5 × 10⁵ cells per well) were plated in 6-well plates and incubated at 37 °C for 24 h. They were then exposed to varying concentrations of CCL (0, 50, 100, and 250 μg/mL) for another 24 h. After treatment, the cells were washed, stained with Annexin V-FITC and PI (20 μg/mL), and examined using flow cytometry (Accuri BD Biosciences, San Jose, CA, USA).

### 4.6. Measurement of Intracellular ROS Level

Excessive ROS generation can lead to cellular damage and apoptosis. In this study, ROS level was measured to evaluate oxidative stress induced by CCL in treated cells. Intracellular ROS levels were assessed using DCFH-DA. Cells were plated in 6-well plates (2 × 10⁵ cells per well) and incubated for 24 h. They were then treated with varying concentrations of CCL (0, 50, 100, and 250 µg/mL) for another 24 h. Following treatment, the cells were washed with PBS and incubated in a fresh culture medium containing 25 µM DCFH-DA at 37 °C for 30 min. The cells were then collected and analyzed using flow cytometry (BD Biosciences, Milpitas, CA, USA). Data were processed with BD Accuri C6 Plus software version 264.21.and fluorescence signals were represented as histograms.

### 4.7. Colony Formation Assay

The colony formation, or clonogenic assay, is an in vitro quantitative technique used to evaluate the ability of a single cell to proliferate and form a large colony through clonal expansion [49]. The colony formation assay was performed to assess the long-term proliferative ability of cancer cells following treatment with CCL. MCF-7 and HeLa cells (2000 cells per well) were suspended in a complete growth medium containing designated concentrations of CCL (0, 10, 25, 50, 100, and 250 µg/mL) and incubated for 24 h. The cultures were then maintained for 15 days in a 5% CO_2_ incubator at 37 °C. After incubation, the cells were stained with 0.5% crystal violet, and colonies containing at least 50 cells were observed and counted under a light microscope at 40× magnification.

### 4.8. Migration Assay

Cell migration plays a crucial role in essential physiological processes, including cancer cell dissemination and tumor metastasis. We used a migration assay to assess the effect of CCL on the migratory ability of cancer cells. A migration assay was performed using 6-well plates, where a monolayer of MCF-7 and HeLa cells was scratched to create a wound. The initial scratch was imaged at 0 h, followed by treatment with varying concentrations of CCL (0, 50, 100, and 250 µg/mL) for 48 h. Cell migration was evaluated by measuring the reduction in the scratch width using an inverted microscope. The extent of wound closure was determined by comparing the scratch distance at 0 h and 48 h.

$$\mathrm{Relative}\;\mathrm{closure}\;\mathrm{of}\;\mathrm{the}\;\mathrm{scratch}\;\left(\%\right)=\frac{\mathrm{Distance}\;\mathrm{at}\;0\;h-\mathrm{Distance}\;\mathrm{at}\;48\;h}{\mathrm{Distance}\;\mathrm{at}\;0\;h}\times 100\%$$

### 4.9. Molecular Docking

A molecular docking study was conducted to predict the binding interactions between bioactive compounds from CCL and key target proteins involved in cancer progression by evaluating their binding affinity and molecular interactions. The X-ray crystallographic structure of the BAX protein (PDB: 1F16) was retrieved from the RCSB Protein Data Bank. To prepare the complex, water molecules, ions, and ligands were removed before saving it in .pdb format. The chemical structures of verbascoside, isoverbascoside, and hispidulin were designed using Chem3D Ultra version 8.0 and subsequently converted into three-dimensional (3D) .pdb files via Chem3D Ultra. Molecular docking studies were conducted using AutoDock 4.2, with input files prepared through AutoDockTools v1.5.6 (The Scripps Research Institute, La Jolla, CA, USA). During preparation, atom types were adjusted, water molecules were removed, and polar hydrogen atoms, along with Gasteiger charges, were added. The BAX grid was set to 50 × 50 with coordinates 19.937, 5.318, and 1.086. Structure files were saved in PDBQT format for docking simulations. Molecular docking was performed using AutoDock 4.2, selecting conformations with the best binding energies for further analysis. The docked structures were visualized using Discovery Studio software version 21.1.0.20298.

### 4.10. Statistical Analysis

The normality distribution of the data was tested using the Shapiro–Wilk test prior to statistical analysis. Statistical analysis of the data was conducted using a two-way ANOVA, followed by Tukey’s multiple comparison test as a post-hoc analysis to ascertain the significance of the observed differences. Non-parametric statistics were applied as appropriate. GraphPad Prism 7.02 software (La Jolla, CA, USA) was employed for this purpose. A significance threshold of *p* < 0.05 was applied uniformly across all analyses to denote statistical significance.

## 5. Conclusions

This study demonstrates the potent anticancer effects of *Clerodendrum chinense* leaf extract (CCL) against MCF-7 breast cancer and HeLa cervical cancer cells. CCL exerted cytotoxic activity in a time- and dose-dependent manner, inducing late apoptosis and necrosis, increasing reactive oxygen species (ROS) generation, and inhibiting colony formation and cell migration. Molecular docking studies revealed strong interactions between CCL bioactive compounds and the pro-apoptotic BAX protein, supporting their role in apoptosis induction. These findings pave the way for developing CCL-based therapies as an affordable and natural alternative for cancer treatment.

## Figures and Tables

**Table 1:** The contents of verbascoside, isoverbascoside, and hispidulin in CCL (*n* = 3).

Samples	Contents (µg/mL)					%RSD
	A	B	C	Mean	SD	
Verbascoside	926.44	877.32	897.96	900.57	24.6657	2.74
Isoverbascoside	266.92	254.16	256.57	259.22	6.7769	2.61
Hispidulin	66.20	63.52	64.15	64.62	1.4017	2.17

**Table 2:** IC_50_ values (µg/mL) of CCL and its bioactive compounds against cancer cells.

Cells	Samples	24 h	48 h	72 h
MCF-7	Hispidulin	905.2	710.4	527.2
	Verbascoside	185.6	183.6	173.3
	Isoverbascoside	113.2	173.8	156.2
	CCL	425.6	192.2	126.8
HeLa	Hispidulin	98.18	119.1	311
	Verbascoside	372.6	290.8	341.8
	Isoverbascoside	377	299.2	376.3
	CCL	196.7	152.7	216.1
RAW 264.7	Hispidulin	99.18	120.4	180.2
	Verbascoside	431.7	445.9	480.2
	Isoverbascoside	499.1	535	652
	CCL	542.7	151	195.3

**Table 3:** The toxicity comparison ratios of CCL and its bioactive compounds against cancer cells.

Cell Lines	Samples	24 h	48 h	72 h
MCF-7	Hispidulin	0.1	0.2	0.3
	Verbascoside	2.3	2.4	2.8
	Isoverbascoside	4.4	3.1	4.2
	CCL	1.3	0.8	1.5
HeLa	Hispidulin	1.0	1.0	0.6
	Verbascoside	1.2	1.5	1.4
	Isoverbascoside	1.3	1.8	1.7
	CCL	2.8	1.0	0.9

**Table 4:** Molecular docking of verbascoside, isoverbascoside, and hispidulin with BAX.

PDB	At Trigger Site	Grid Map			
1F16	Docking 50 Runs	X	Y	Z	Grid Size
		19.937	5.318	1.086	50 × 50 × 50
**Compound**		**Binding Energy (Kcal/mole)**	**Ki (uM)**	**Contacted Amino Acid**	
BAXactivator (BTC-8)		−7.64	2.53	PRG8, ARG9, GLY10, GLY11, GLY12, PRO13, THR14, SER15, SER16, LEU149, GLN153	
Verbascoside		−4.48	515.93	PRG8, ARG9, GLY12, PRO13, THR14, SER15, SER16, ARG145, GLU146, LEU149, GLY150, GLN153	
Isoverbascoside		−4.94	239.56	PRG8, ARG9, GLY10, GLY11, GLY12, PRO13, THR14, SER15, SER16, MET20, ARG145, LEU149, GLY150, GLN153	
Hispidulin		−6.35	22.00	PRG8, ARG9, GLY10, GLY11, GLY12, PRO13, THR14, SER15, SER16, LEU149, GLY150, GLN153	

## Data Availability

Data are available only in this article.
